# Molecular detection of free-living amoebae from Namhangang (southern Han River) in Korea

**DOI:** 10.1038/s41598-019-57347-1

**Published:** 2020-01-15

**Authors:** Heekyoung Kang, Hae-Jin Sohn, Ga-Eun Seo, Gi-Sang Seong, A-Jeong Ham, A-Young Park, Suk-Yul Jung, Sang-Eun Lee, Shin-Hyeong Cho, Ho-Joon Shin

**Affiliations:** 1 Department of Microbiology, Ajou University School of Medicine, Suwon, 16499 Republic of Korea; 2 Department of Biomedical Science, Graduate School of Ajou University, Suwon, 16499 Republic of Korea; 3 Department of Biomedical Laboratory Science, Molecular Diagnostics Research Institute, School of Health and Medicine, Namseoul University, Cheonan, 31020 Republic of Korea; 4 Division of Vectors and Parasitic Diseases, Korea Centers for Diseases Control and Prevention, Osong, 363-951 Republic of Korea

**Keywords:** Molecular ecology, Parasite biology

## Abstract

The free-living amoebae *Naegleria* spp. and *Acanthamoeba* spp. exist in the natural environment and are sometimes causal agents of lethal primary amoebic meningoencephalitis (PAM), amoebic keratitis (AK) and granulomatous amebic encephalitis (GAE) in humans, respectively. To ascertain the existence of free-living amoebae in Korea, water samples were collected from the Korean hydrosphere, Namhangang (southern Han River), an active location for water skiing and recreation. Samples underwent two-step filtration and were cultured on non-nutrient agar medium with inactivated *E. coli*. The remaining samples were subjected to PCR for primarily the 18S small ribosomal RNA gene and gene sequencing. Similarities in 18S rDNA sequences, in comparison with various reference amoebae in GenBank, showed 86~99% homology with *N. gruberi*, *N. philippinensis*, *N. clarki*, *A. polyphaga, A. castellannii*, and *Hartmannella* (*Vermamoeba*) *vermiformis*. Therefore, this study will be useful for seasonal detection of free-living amoebae from various Korean hydrospheres in future studies.

## Introduction

The free-living amoebae (FLA) *Naegleria* spp. and *Acanthamoeba* spp. are mainly distributed in ponds, rivers, and fresh waters worldwide. Their existing stages are trophozoite and cyst and additional biflagellate form in in case of *Naegleria*. Trophozoites shows moving, feeding, and proliferation activity. However, cysts are formed in poor environments, such as under abrupt temperature changes, drying, and food depletion, and can survive for long periods^1,2^. *N. fowleri* is a pathogenic agent that causes primary amoebic meningoencephalitis (PAM), which is fatal to humans and laboratory animals^2,3^. *Acanthamoeba* spp. and *Balamuthia mandrillaris* cause chronic granulomatous amebic encephalitis (GAE)^4–6^. Further, *A. castellanii* and *A. polyphaga* can infect the eye, resulting in acanthamoeba keratitis (AK)^2,7,8^.

PAM is mainly associated with activities in amoeba-contaminated water (swimming or water leisure activity), use of Neti-pots in rhinitis treatment, and religious ceremonies in some Asian countries^5,9,10^. The amoeba enters through the nasal cavity to invade the mucosal membrane. Subsequently, it moves into the olfactory bulb and meninges via the nasal nerve system, leading to development of meningoencephalitis^11–13^. Symptoms of PAM include acute headache, anorexia, nausea, vomiting, high fever (38–40 °C), and limb dysfunction symptoms. It also progresses acutely, with a mortality rate of over 95%. Amphotericin B is mainly used as a therapeutic agent, as a combination treatment by mixing with micronazole, rifampin, and doxycycline; however, only limited therapeutic effects have been demonstrated^1,2,14–16^.

AK usually occurs after wearing contaminated contact lenses, improper ophthalmic surgery, or corneal injury in various cases. With the popularization of contact lenses and careless lens management, the number of AK patients continues to increase^2,7,17,18^.

PAM due to *N. fowleri* occurs annually worldwide. In the United States, 143 cases of PAM occurred from 1962 to 2017, of which 80% were in males. Further, infection was most reported in adolescents^19–22^. In Pakistan, 22 patients in 2012 were found to be infected with *N. fowleri* after washing their nostrils with tap water as a religious ceremony^10,23^. Additional PAM patients are expected worldwide. In Korea, there has been no case of PAM caused by *N. fowleri*; yet, one case of GAE due to *Acanthamoeba* sp. has been reported^24^. Conversely, multiple AK patients have been identified and reported^25–28^.

The occurrence of PAM cases continues to increase in the tropics and subtropics, with increasing risk worldwide due to global warming. In addition, as many people enjoy water-related sports, PAM occurrence has been a social issue through the broadcast media. While it has been reported in many countries, there has not yet been reported in South Korea. There, we wanted to find out what kinds of free living amoeba, especially *N. fowleri*, exist in South Korea. In addition, various FLAs are expected to reside in the Korean hydrosphere. Therefore, a distribution survey was conducted to identify *Naegleria* and *Acanthamoeba* species in southern Han River, an active location for water skiing and recreation.

## Materials and Methods

### Water sample collection

**Figure 1:**
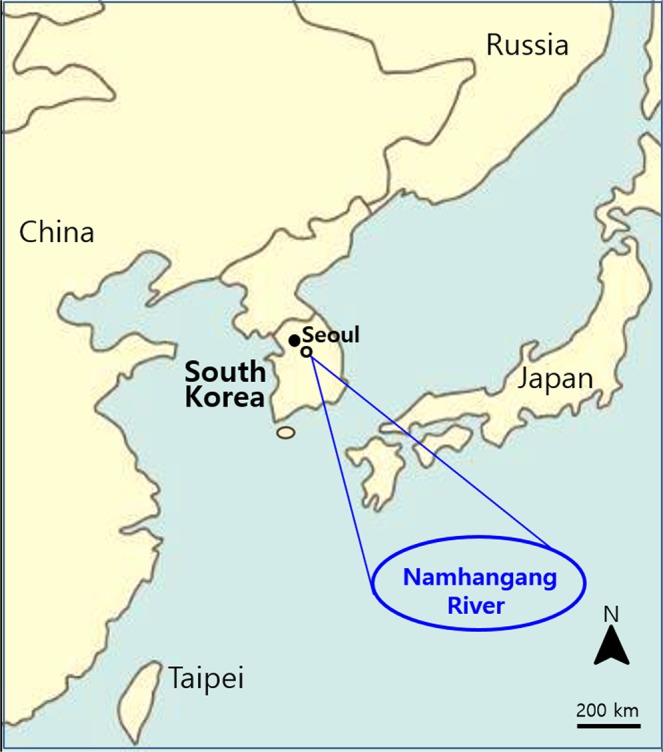
Location of Namhangang River (nearby Yeoju city and Yangpyeong) in South Korea. Water sampling from Namhangang, location where water skiing and recreation are actively performed. Maps made by H-J Shin in Adobe photoshop (version 7.0.1, https://www.adobe.com/).

**Figure 2:**
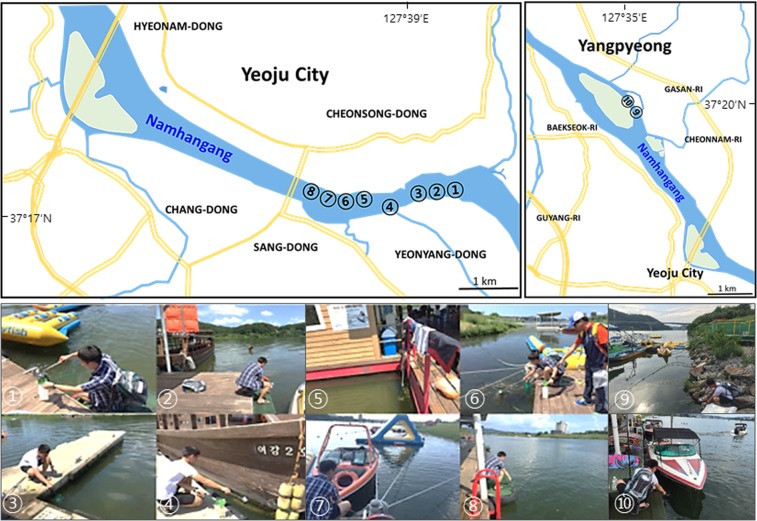
Water collection sites in Yeoju city (①~⑧) and Yangpyeong (⑨,⑩). The collection was repeated at the same sites for one year. Maps made by H-J Shin in Adobe photoshop (version 7.0.1, https://www.adobe.com/). Photograph by H-J Sohn.

To investigate the distribution of free living amoeba in Yeoju city and Yangpyeong-gun, near southern Han River (Namhangang), which are geographically close to Seoul, we collected 1-L surface water samples with a 20-cm diameter scoop from 10 sites periodically from August 2015 to August 2016 (over 120 water samples) (Fig. 1). Sample collection sites where carries out water recreational activities were shown in Fig. 2. The atmospheric temperature at the water sampling sites was between −11 °C and 35 °C, and the water temperature ranged from 0.1 °C to 29 °C, especially 26–29 °C in August (Table 1).

**Table 1:** Summary of free-living amoebae and other microorganisms from Yeoju and Yangpyeong water samples identified by the homology analysis with 18s-rRNA gene sequence.

Collection date	Identified free-living amoebae and microorganisms	Homology (%)	Ambient temp. (°C) (Mini/Max)	Water temp. (°C)
August 2015	*Naegleria clarki*	97%	20/35	26~29
	*Naegleria gruberi*	99%		
	*Acanthamoeba castellanii*	99%		
	*Acanthamoeba polyphaga*	98%		
	*Naelgeria philippinensis*	99%		
	*Naegleria australiensis*	95%		
	*Acanthamoeba lugdunensis*	99%		
	*Hartmannella* (*Vermamoeba*) *vermiformis*	97%		
	*Scapholeberis mucronata*	—		
	Uncultured fungus	—		
September	*Tetmemena* sp.	—	11/27	23~24
	*Stichotrichia* sp.	—		
	*Brachionus plicatilis*	—		
	*Oxytricha ferruginea*	—		
	Uncultured ciliate	—		
	Uncultured eukaryote	—		
October	*Thalassiosira gravida*	—	7/25	17~19
	*Scapholeberis mucronate*	—		
	*Choreotrichia* sp.	—		
	Uncultured metazoan	—		
	Uncultured eukaryote	—		
	Uncultured fungus	—		
November	*Naegleria gruberi*	86%	6/14	13~14
	*Naegleria clarki*	98%		
	*Paramecium pytrinum*	—		
	*Vorticella fusca*	—		
	*Choreotrichia* sp.	—		
	Uncultured ciliate	—		
	Uncultured eukaryote	—		
December	*Stephanodiscus parvus*	—	−10/−2	0.1~4.2
	*Skeletonema subsalsum* strain	—		
	*Skeletonema costatum*	—		
	Uncultured eukaryote	—		
January 2016	*Stephanodiscus parvus*	—	−11/−1	0.8~2.1
	*Paramecium putrinum*	—		
	*Synura petersenii*	—		
	*Synura glabra*	—		
	Uncultured eukaryote	—		
February	*Spumella* sp.	—	−5/8	3.3~4.7
	Uncultured eukaryote	—		
March	*Mallomonas cratis*	—	−2/17	8.6~12
	*Mallomonas tonsurata*	—		
	*Odontella rostrata*	—		
	S*ynura* sp.	—		
	Uncultured eukaryote	—		
	Uncultured ciliate	—		
April	*Stephanodiscus* sp.	—	9/19	12~18
	Uncultured eukaryote	—		
May	*Peridiniopsis penardii*	—	13/25	17~19
	*Discostella stelligera* strain	—		
	*Hatschekia japonica*	—		
	*Thalassiosira* sp.	—		
	Uncultured eukaryote	–—		
June	*Lacinularia flosculosa* isolate	—	17/30	24~26
	*Selaginella wildenowii*	—		
	Uncultured ciliate	—		
	Uncultured eukaryote	—		
July	*Tintinnidium balechi* isolate	—	23/33	26~28
	*Cricetulus griseus*	—		
	*Choreotrichia* sp.	—		
	Uncultured eukaryote	—		

### Harvesting of FLA

**Figure 3:**
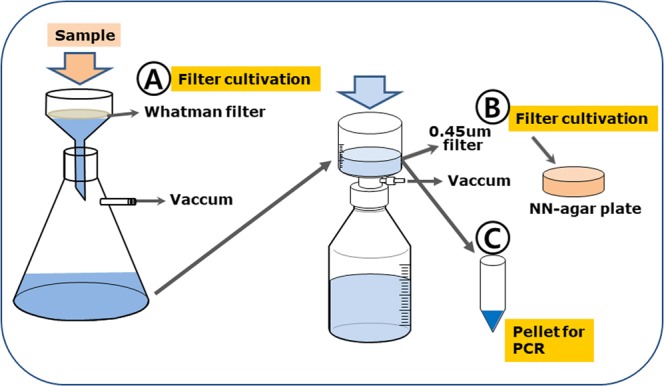
Free-living amoebae collection methods using two-filtration system. After the second filtration, a filter and some suspended water were cultured on NN-agar medium (**B**), and the remaining water pellet was subjected to PCR for 18s-rRNA gene amplification. The figure was prepared by H-J Shin using Adobe photoshop (version 7.0.1, https://www.adobe.com/).

To concentrate FLA from water samples, we used a two-step filtration system. The collected surface water was first filtered using a Whatman filter, followed by filtration (with a 0.4 μm pore-size bottle top filter (Thermo Fisher Scientific Inc., USA) (Fig. 3). After leaving about 30 mL of solution and thorough washing of the filter, the mixture was centrifuged at 1,500 rpm for 5 min. A portion of the pellet and filter paper were placed on a non-nutrient agar (NN-agar) plate for the amoeba culture, and the remaining pellet was subjected to PCR for amplification of the 18S RNA gene.

### Culturing of FLA

To culture FLA, the NN-agar plate was prepared with NN-agar medium. Briefly, 15 g of nutrient agar medium and 0.1 g of yeast extract were dissolved in 1,000 mL of distilled water. After sterilization and solidification, the agar medium was poured into 10 petri dishes. *Escherichia coli* was killed at 60 °C for 30 min, and then evenly coated on the surface of the prepared agar plate. The prepared specimen was dropped onto the medium, following which the culture dish was covered and incubated at 27 °C for 2–4 days. The amoebae were sub-cultured on fresh NN-agar plates two or three times. Further, *N. fowleri*, *A. castellanii*, and *A. polyphaga* used as positive control amoebae were cultured on Nelson’s and PYG medium according to previous reports^29,30^. The morphological identification of cultured amoebae was observed with an inverted microscope (Olympus CKX 31, Japan).

### PCR identification of FLA

DNA was extracted from environmental sample according to the manufacturer’s protocol (Qiagen, USA). Next, molecular identification of FLA has been performed as described below.

Briefly, 2 uL of the extracted DNA was used as a template, and mixed with 10 μL of Noblezyme ™ PCR Plus Premix (Noble Bioscience Inc., Korea) in a PCR tube. Primer pairs to amplify the 18S rRNA gene of various FLAs, called pan primers (P-FLA) (2pi, Bioneer Inc., Korea), were according to previous studies^31,32^. The recommended amplicon sizes were as follows: *Acanthamoeba* spp., 1080–1500 bp; *Vahlkampfia* spp., 950 bp; *N. fowleri*, 900 bp; and *Vermamoeba (Hartmannella) vermiformis*, 800 bp. Amplification was performed with an initial polymerase activation step (5 min at 95 °C), followed by 35 cycles of denaturation (1 min s at 94 °C), primers hybridisation (1 min at 60 °C), and extension (3.5 min at 74 °C) in a G-Storm thermocycler (Genetechnologie, UK). All PCR experiments were performed with the inclusion of positive controls (DNA extracted from *N. fowleri*, *A. polyphaga*, and *A. castellanii*) to ensure correct functionality of the reaction.

After completion of the reaction, the PCR products were electrophoresed at 120 V for 20 min using a 1% agarose gel stained with ethidium bromide (0.005%) and then analyzed by Gel-doc (Bio-rad, USA).

### 18S rDNA sequencing and phylogenetic analysis

The FLA nucleotide sequences of the PCR-amplified 18S rRNA gene were obtained from the direct sequencing (Genotech, Daejeon, Korea), and homology against registered FLAs in GenBank was analyzed. Based on this, we conducted FLA phylogenetic analysis by estimating the neighbor-joining distance using the MEGA6 program^33^.

## Results

### Morphology of cultured FLA

**Figure 4:**
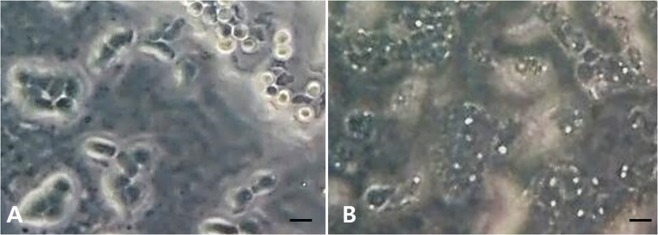
Free-living amoebae cultured on non-nutrient agar plate, predicted as *Naegleria* sp. (**A**) and *Acanthamoeba* sp. (**B**). Bars, 10 μm.

FLA cultured from water samples showed a resemblance to the presumed genus *Naegleria* or *Acanthamoeba*, as trophozoites showing round pseudopodia or acanthopodia; cysts were also observed in the colony (Fig. 4).

### 18S rRNA gene sequence and alignment

**Figure 5:**
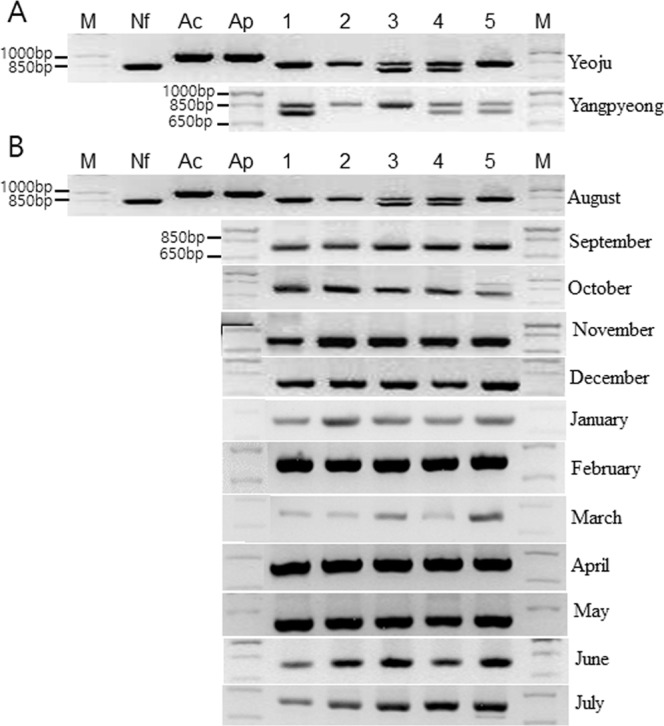
Amplified PCR products using P-FLA primers in water samples collected from Nanhangan, Yeoju city, and Yangpyeong (**A**), and a year in Yeoju city (**B**). Nf; *N. fowleri*, Ac; *A. castellanii*, Ap; *A. polyphaga*; lane 1–5, water sampling sites. M; PCR molecular marker.

**Figure 6:**
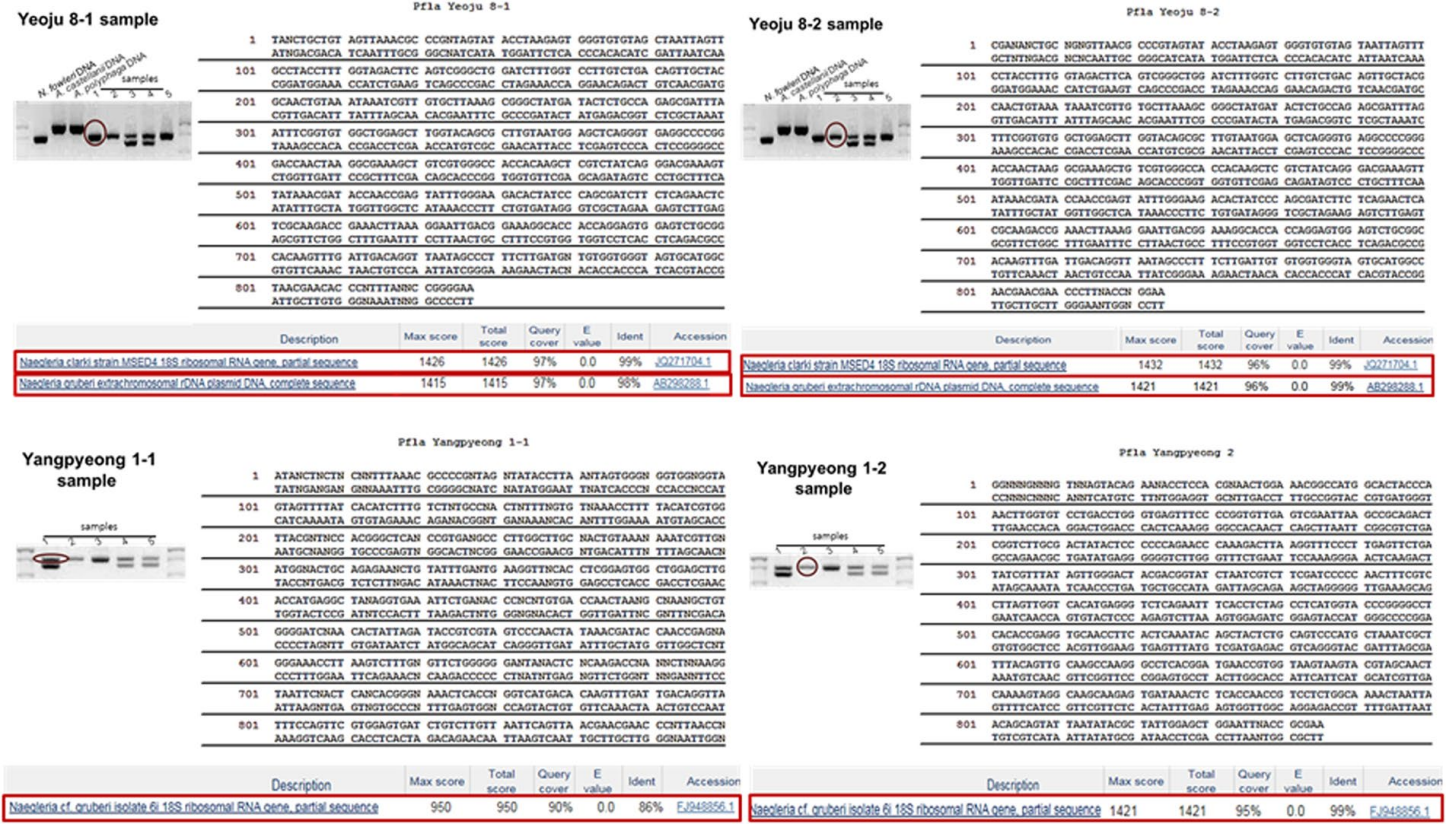
18S rDNA sequences and homology search of FLA isolates in Yeoju city and Yangpyeong.

**Figure 7:**
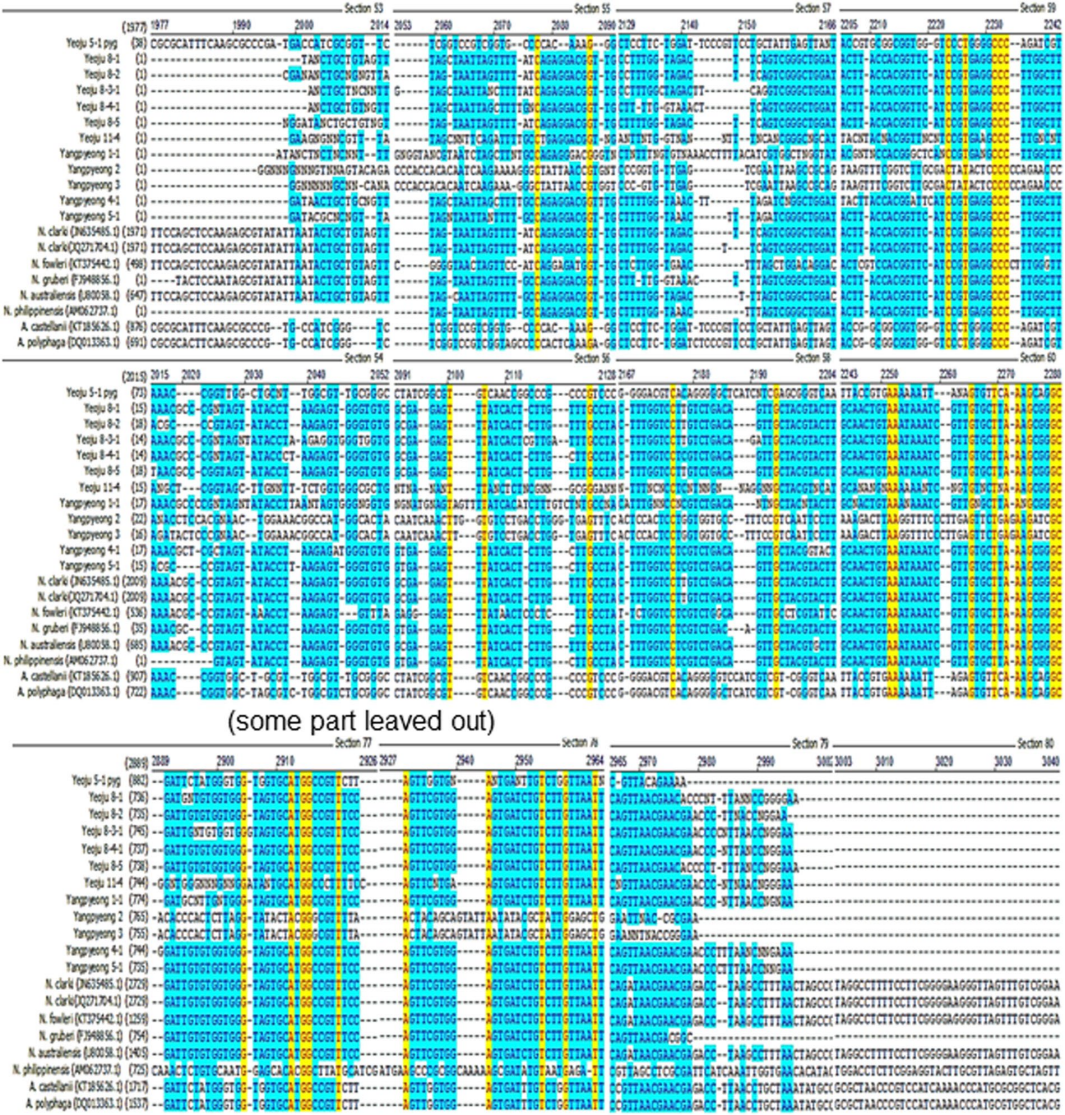
Alignment of 18s-rDNA sequences of free-living amoebae isolated from Namhangang against the reference amoeba.

Based on the PCR results using P-FLA primers, the main PCR reaction bands were 700–900 bp for Yeoju water samples collected throughout the year (Fig. 5). Further, a homology search of DNA sequences from 18S rRNA genes of FLA isolates indicated matches with *N. gruberi* (99%), *N. philippinensis* (99%), *N. clarki* (97%), *A. polyphaga* (98%)*. A. castellannii* (99%), and *Hartmannella* (*Vermamoeba*) *vermiformis* (97%), as which one Yangpyeong sample showed the lowest homology with *N. gruberi* (86%), and one Yeoju sample showed the highest homology with *N. clarki* (99%) by sequence alignment (Figs. 6 and 7). And then, summary of results was showed in Table 1.

### Phylogenetics of FLA 18S rDNA sequences

**Figure 8:**
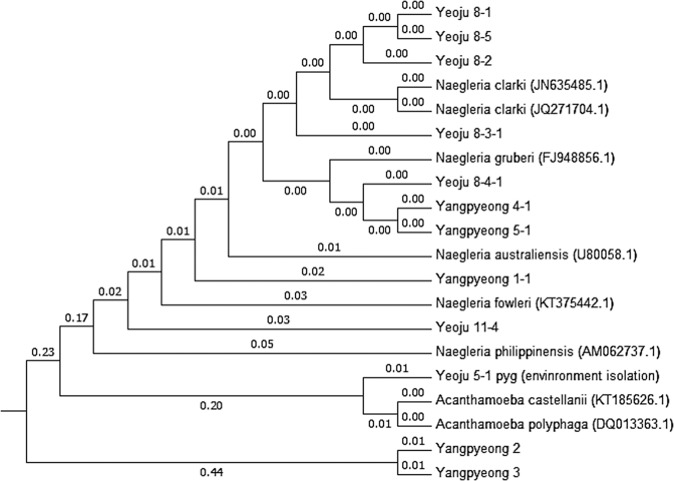
Estimation of neighbor-joining distance tree by phylogenetic analysis of free-living amoebae based on 18s-rDNA sequences.

A neighbor-joining distance tree was constructed based on phylogenetic analysis of the DNA sequences from the amplified FLA 18S rRNA gene. Many Yeoju specimens clustered mainly with *N. clarki* and *N. gruberi*, whereas Yangpyeong samples were closely related to *N. gruberi* and *N. australiensis* (Fig. 8). However, one of the Yeoju samples clustered with *A. castellanii* and *A. polyphaga* (Fig. 8).

## Discussion

Epidemiological studies of PAM have mainly occurred in the southeastern part of the United States, through swimming and watering activities in *N. fowleri*-contaminated freshwater, such as lakes and ponds, during the summer months^20,34,35^. Subsequently, in other countries, various survey have been conducted on ponds, lakes, rivers, swimming pools, hot springs, and contaminated sewage, which are known as FLA habitats.

In Thailand and Japan, *Naegleria* and *Acanthamoeba* species were detected in hot springs frequented by tourists and local residents^36,37^. In Italy, New Zealand, and California of the United States, *N. fowleri* was detected in a river area used for swimming and in a swimming pool^38–40^. In Taiwan, the nearest Asian country, *N. fowleri* was isolated from hot springs, and *Acanthamoeba* and *Naegleria* spp. from recreational water^41^. *Acanthamoeba* spp. was detected in water in neighboring China^42^. In Korea, *Acanthamoeba* spp. has been detected in amoeba-contaminated tap water in damaged water pipes and in several AK patients^43,44^.

We investigated only water samples where water sports or leisure activities were active, because we have the interest in public health which is caused by pathogenic free-living amoebae, especially *Naegleria* spp. and *Acanthamoeba* spp. Based on this survey on FLA distribution in the Korean hydrosphere, especially Namhangang, various species of *Naegleria* sand *Acanthamoeba* were found in Yeoju and Yangpyeong samples, especially in August. Notably, the highly virulent *N. fowleri* (which favors high temperatures) was not found in this survey, possibly because the temperature of the water system was inadequate (measured as 26–29 °C). A future detailed and extensive survey will be required to determine whether virulent *N. fowleri* inhabits this area. We conducted the survey over the course of a year to observe seasonal variations as well. However, the temperature in Korea sharply decreased in September, and the river surface water was frozen in December. As such, we could not observe FLA from September to June, although some bacteria and fungi were detected (data not shown).

Because many studies have not described the detailed process of FLA collection in an environmental water system, especially in rivers containing many floats, the culture protocol using NN-agar medium and the PCR protocol were difficult to conduct. By implementing the two-filtration system, we were able to overcome this limitation. After two filtrations, we were able to readily perform the amoeba culture and obtain PCR results. In addition, we attempted aseptic culture with Nelson’s and PYG medium, which are commonly used in *Naegleria* and *Acanthamoeba* culture protocols. However, much time and effort are required for successful aseptic culture. Several *Acanthamoeba* species were successfully cultured; however, no *Naegleria* spp. could be cultured aseptically. These results will be published in a future report.

On the results of PCR-based DNA amplification, amoebic 18S rRNA sizes amplified with PAN primer, especially *Acanthamoeba* species, from water samples and reference amoebae were smaller than that suggested in previous paper^31,32^. Although there were differences in size, sequencing results were consistent and complete in this study. This issue is worth further study. Another primer, Nfa1 and ITS primer for the amplification of *Naegleria* spp.^29^, and 18s-rDNA primer for *Acanthamoeba* spp.^27^, was applied in the preliminary experiment, but various amoebae were not amplified (data not shown). Otherwise, PAN primer amplified various species.

Considering that FLA was isolated in many countries worldwide, especially around the Korean peninsula that PAM cases or *N. fowleri* were detected in the natural environment in China and Japan, the survey of FLA distribution in the Korean hydrosphere was required. As suggested in this study, the discovery of pathogenic *Acanthamoeba* sp. in southern Han River poses a potential risk to Korea. Therefore, future studies are necessary to investigate its wide distribution in various water environments, such as rivers with frequent water activities, hot springs, and swimming pools, in Korea.

## References

[1] Ma P (1990). *Naegleria* and *Acanthamoeba* infections: review. Rev. Infect. Dis..

[2] Visvesvara GS, Moura H, Schuster FL (2007). Pathogenic and opportunistic free-living amoebae: *Acanthamoeba* spp., *Balamuthia mandrillaris*, *Naegleria fowleri*, and *Sappinia diploidea*. FEMS Immunol. Med. Microbiol..

[3] Carter RF (1970). Description of a *Naegleria* sp. isolated from two cases of primary amoebic meningo-encephalitis, and of the experimental pathological changes induced by it. J. Pathol..

[4] Schafer KR (2015). Disseminated *Balamuthia mandrillaris* Infection. J. Clin. Microbiol..

[5] Schuster FL, Visvesvara GS (2004). Free-living amoebae as opportunistic and non-opportunistic pathogens of humans and animals. Int. J. Parasitol..

[6] Sutcu M (2018). Granulomatous amebic encephalitis caused by Acanthamoeba in an immuncompetent child. Turk. J. Pediatr..

[7] Alfonso-Munoz EA (2018). A report of 10 patients with Acanthamoeba keratitis. Arch. Soc. Esp. Oftalmol..

[8] Auran JD, Starr MB, Jakobiec FA (1987). Acanthamoeba keratitis. A review of the literature. Cornea.

[9] Ghanchi NK (2017). Case Series of *Naegleria fowleri* Primary Ameobic Meningoencephalitis from Karachi, Pakistan. Am. J. Trop. Med. Hyg..

[10] Siddiqui, R. & Khan, N. A. Primary amoebic meningoencephalitis caused by *Naegleria fowleri*: an old enemy presenting new challenges. *PLoS Negl Trop Dis* **8**, e3017, 0003017 (2014). 10.1371/journal.pntd.0003017 PMC4133175 25121759

[11] Carter RF (1972). Primary amoebic meningo-encephalitis. An appraisal of present knowledge. Trans. R. Soc. Trop. Med. Hyg..

[12] Jarolim KL, McCosh JK, Howard MJ, John DT (2000). A light microscopy study of the migration of *Naegleria fowleri* from the nasal submucosa to the central nervous system during the early stage of primary amebic meningoencephalitis in mice. J. Parasitol..

[13] John DT (1982). Primary amebic meningoencephalitis and the biology of *Naegleria fowleri*. Annu. Rev. Microbiol..

[14] Jain R, Prabhakar S, Modi M, Bhatia R, Sehgal R (2002). Naegleria meningitis: a rare survival. Neurol. India.

[15] Seidel JS (1982). Successful treatment of primary amebic meningoencephalitis. N. Engl. J. Med..

[16] Wang Q (2018). A case of *Naegleria fowleri* related primary amoebic meningoencephalitis in China diagnosed by next-generation sequencing. BMC Infect. Dis..

[17] Fears AC, Metzinger RC, Killeen SZ, Reimers RS, Roy CJ (2018). Comparative *in vitro* effectiveness of a novel contact lens multipurpose solution on *Acanthamoeba castellanii*. J. Ophthalmic Inflamm. Infect..

[18] Lorenzo-Morales J, Khan NA, Walochnik J (2015). An update on Acanthamoeba keratitis: diagnosis, pathogenesis and treatment. Parasite.

[19] Matanock A, Mehal JM, Liu L, Blau DM, Cope JR (2018). Estimation of Undiagnosed *Naegleria fowleri* Primary Amebic Meningoencephalitis, United States (1). Emerg. Infect. Dis..

[20] Centers for Disease Control and Prevention-USA *Naegleria fowleri*–case report data & graphs. [insert website/webpage name here], https://www.cdc.gov/parasites/naegleria/graphs.html#case-reports, Accessed [insert date here] (2019)

[21] Visvesvara GS, Stehr-Green JK (1990). Epidemiology of free-living ameba infections. J. Protozool..

[22] Yoder JS, Eddy BA, Visvesvara GS, Capewell L, Beach MJ (2010). The epidemiology of primary amoebic meningoencephalitis in the USA, 1962–2008. Epidemiol. Infect..

[23] Shakoor S (2011). Primary amebic meningoencephalitis caused by *Naegleria fowleri*, Karachi, Pakistan. Emerg. Infect. Dis..

[24] Ringsted J, Jager BV, Suk D, Visvesvara GS (1976). Probable acanthamoeba meningoencephalitis in a Korean child. Am. J. Clin. Pathol..

[25] Kim EC, Kim MS (2010). Bilateral acanthamoeba keratitis after orthokeratology. Cornea.

[26] Lee JE (2007). Acanthamoeba keratitis related to orthokeratology. Int. Ophthalmol..

[27] Xuan YH (2008). Keratitis by *Acanthamoeba triangularis*: report of cases and characterization of isolates. Korean J. Parasitol..

[28] Lee JK, Lee JS (2015). Two Cases of Corneal Toxicity in Acanthamoeba Keratitis by Combined Topical Anti-Acanthamoeba Keratitis Eye Solution. J. Korean Ophthalmol. Soc..

[29] Kang H (2015). Effective PCR-based detection of *Naegleria fowleri* from cultured sample and PAM-developed mouse. Eur. J. Protistol..

[30] Sohn HJ (2017). Efficient Liquid Media for Encystation of Pathogenic Free-Living Amoebae. Korean J. Parasitol..

[31] Schroeder JM (2001). Use of subgenic 18S ribosomal DNA PCR and sequencing for genus and genotype identification of acanthamoebae from humans with keratitis and from sewage sludge. J. Clin. Microbiol..

[32] Tsvetkova N (2004). The identification of free-living environmental isolates of amoebae from Bulgaria. Parasitol. Res..

[33] Tamura K, Stecher G, Peterson D, Filipski A, Kumar S (2013). MEGA6: Molecular Evolutionary Genetics Analysis version 6.0. Mol. Biol. Evol..

[34] De Jonckheere JF (2011). Origin and evolution of the worldwide distributed pathogenic amoeboflagellate *Naegleria fowleri*. Infect. Genet. Evol..

[35] Heggie TW (2010). Swimming with death: *Naegleria fowleri* infections in recreational waters. Travel. Med. Infect. Dis..

[36] Izumiyama S (2003). Occurrence and distribution of *Naegleria* species in thermal waters in Japan. J. Eukaryot. Microbiol..

[37] Lekkla A, Sutthikornchai C, Bovornkitti S, Sukthana Y (2005). Free-living ameba contamination in natural hot springs in Thailand. Southeast. Asian J. Trop. Med. Public. Health.

[38] Cursons R, Sleigh J, Hood D, Pullon D (2003). A case of primary amoebic meningoencephalitis: North Island, New Zealand. N. Z. Med. J..

[39] Johnson RO (2016). Notes from the Field: Primary Amebic Meningoencephalitis Associated with Exposure to Swimming Pool Water Supplied by an Overland Pipe - Inyo County, California, 2015. MMWR Morb. Mortal. Wkly. Rep..

[40] Montalbano Di Filippo M, Novelletto A, Di Cave D, Berrilli F (2017). Identification and phylogenetic position of *Naegleria* spp. from geothermal springs in Italy. Exp. Parasitol..

[41] Tung MC (2013). Identification and significance of *Naegleria fowleri* isolated from the hot spring which related to the first primary amebic meningoencephalitis (PAM) patient in Taiwan. Int. J. Parasitol..

[42] Lass A (2017). Detection of *Acanthamoeba* spp. in water samples collected from natural water reservoirs, sewages, and pharmaceutical factory drains using LAMP and PCR in China. Sci. Total. Env..

[43] Jeong HJ, Yu HS (2005). The role of domestic tap water in *Acanthamoeba* contamination in contact lens storage cases in Korea. Korean J. Parasitol..

[44] Kong HH (2009). Molecular phylogeny of *acanthamoeba*. Korean J. Parasitol..

